# MIG-seq: an effective PCR-based method for genome-wide single-nucleotide polymorphism genotyping using the next-generation sequencing platform

**DOI:** 10.1038/srep16963

**Published:** 2015-11-23

**Authors:** Yoshihisa Suyama, Yu Matsuki

**Affiliations:** 1 Tohoku University, Kawatabi Field Science Center, Graduate School of Agricultural Science, 232-3 Yomogida, Naruko-onsen, Osaki, Miyagi 989-6711, Japan

## Abstract

Restriction-enzyme (RE)-based next-generation sequencing methods have revolutionized marker-assisted genetic studies; however, the use of REs has limited their widespread adoption, especially in field samples with low-quality DNA and/or small quantities of DNA. Here, we developed a PCR-based procedure to construct reduced representation libraries without RE digestion steps, representing *de novo* single-nucleotide polymorphism discovery, and its genotyping using next-generation sequencing. Using multiplexed inter-simple sequence repeat (ISSR) primers, thousands of genome-wide regions were amplified effectively from a wide variety of genomes, without prior genetic information. We demonstrated: 1) Mendelian gametic segregation of the discovered variants; 2) reproducibility of genotyping by checking its applicability for individual identification; and 3) applicability in a wide variety of species by checking standard population genetic analysis. This approach, called multiplexed ISSR genotyping by sequencing, should be applicable to many marker-assisted genetic studies with a wide range of DNA qualities and quantities.

The recent development of next-generation sequencing (NGS) technology has allowed the effective discovery and genotyping of large numbers of genome-wide genetic markers^1^. However, many marker-assisted studies require more economical and efficient approaches, rather than the methods based on the high marker density produced by whole-genome sequencing. To optimize the cost and the amount of available information for these studies, several methods have been developed to construct reduced representation libraries (RRLs), to sample single-nucleotide polymorphisms (SNPs) from genome-wide regions, and to genotype them using NGS. Methods such as reduced representation shotgun^2^ sequencing and restriction site-associated DNA (RAD)^3^ markers were later adapted for NGS-based methods by the sequencing of RRLs^4^ and RAD tags^5^. Many improved approaches have been developed in recent years, such as complexity reduction of polymorphic sequences^6^, multiplexed shotgun sequencing^7^, genotyping by sequencing (GBS)^8^, 2-enzyme GBS^9^, RAD genotyping using type IIB restriction enzymes^10^, double digest RAD^11^, and restriction fragment sequencing^12^. These methods have become widespread and allow marker-assisted genetic studies, such as ecological, evolutionary, phylogeographic, and genetic mapping studies, based on tens to hundreds of thousands of SNPs in hundreds of barcoded samples at the same time. However, a more simple, rapid and cost-efficient approach for smaller-scale studies is desired, especially in ecological laboratories.

The above methods use restriction enzymes (REs) to produce a reduced representation of a genome. Therefore, relatively large amounts (normally hundreds of ng per sample, see ref. 1) of high-quality genomic DNA are required for the digestion steps as an absolute condition of the starting materials. These assumptions often limit their adoption for many marker-assisted genetic studies of wild populations in non-model species. For example, sufficient amounts of DNA would be unavailable from not only small-sized organisms, such as microorganisms, but also larger sized species, such as endangered alpine plants, from which only small parts of tissues are allowed to be sampled. High-quality DNA represents a limitation for field samples. Especially in ecological and conservation genetics, sometimes there is no choice but to collect materials from small amounts of degraded tissues. As a consequence, RE-based NGS methods cannot be applied in the fields of ecological and conservation genetics because of the limitation of materials with acceptable standards in DNA quality and quantity.

One possible solution to this problem is to change the RE-based steps to polymerase chain reaction (PCR)-based steps. This is analogous to previous RE-based genetic markers, such as restriction fragment length polymorphisms^13^ and amplified fragment length polymorphisms^14^, which were replaced by PCR-based markers, such as microsatellites^15^ (or simple sequence repeats, SSRs). To amplify sufficient numbers of genome-wide regions adapted for the NGS platform, we selected multiplex PCR primers for inter-simple sequence repeats (ISSRs)^16^,^17^. Although the original method of ISSR analysis detects polymorphisms based on the presence or absence of amplified fragments as dominant markers, we applied ISSR-PCR to amplify a large number of anonymous genome-wide regions without prior genetic information. The 3′ ends of the ISSR primers were designed to anneal to the 3′ ends of repetitive motifs including two bases of anchor sequences and to amplify multiple non-repetitive regions between various SSRs. Our recommended set of multiplex primers included eight primers each for the forward and reverse primers, resulting in 8 × 8 = 64 combinations of primers (Table 1). Although the number of amplified regions depends on the target genome and PCR conditions, thousands of regions were amplified from a wide variety of genomes (see Results), which is effectively a reduced representation library of the genome. Multiplexed PCR amplicons from multiplexed barcoded samples were then sequenced by NGS for SNP detection and genotyping.

Here, we propose a novel approach that we termed “multiplexed ISSR genotyping by sequencing” (MIG-seq), which is a PCR-based procedure for constructing highly reduced representation libraries without RE digestion steps, involving *de novo* SNP discovery, and their genotyping using NGS. To validate the method’s accuracy, we demonstrated: 1) Mendelian gametic segregation of the discovered variants in megagametophyte tissues from a coniferous mother tree of *Picea abies*; 2) the reproducibility of genotyping by checking its applicability in clone (individual) identification of a clonal dwarf bamboo, *Sasa palmata*; and 3) the applicability of the method in a wide variety of species, including plants, animals and fungi, by checking standard population genetic analysis. Although we optimized the procedure for a desktop NGS platform, MiSeq (Illumina, San Diego, CA, USA), it could be applied to other desktop NGS platforms, such as Ion PGM (Thermo Fisher Scientific, Waltham, MA, USA) and GS Junior (F. Hoffmann-La Roche, Basel, Switzerland), with some modifications, or even on large-scale platforms, such as NextSeq 500 and HiSeq (Illumina). The method is applicable to a wide range of marker-assisted genetic studies, especially for medium-scale studies based on fewer than 1,000 markers in ecological, phylogeographic, and conservation genetics, including quick surveys of genetic differentiation among individuals (clones and breeding varieties), populations, closely related species and hybrids.

## Results

### Primer selection and construction of the library

**Figure 1:**
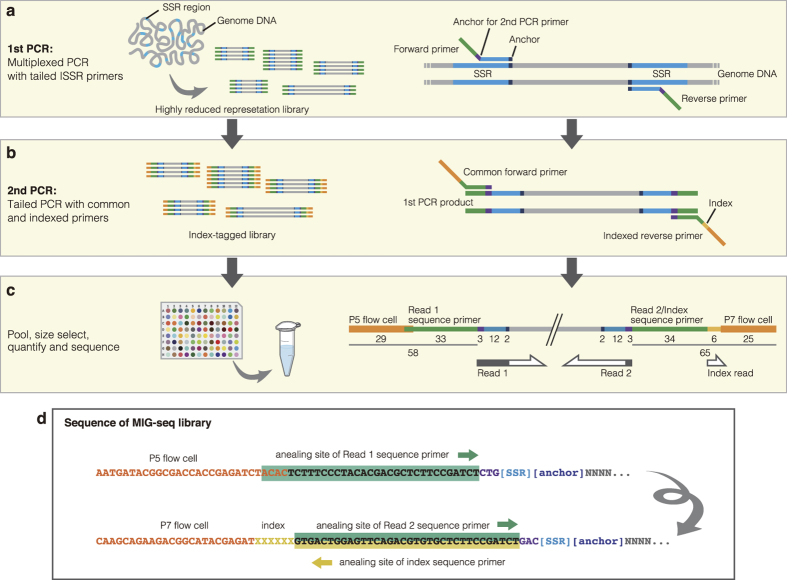
Construction of the MIG-seq library. (**a**) Multiple non-repetitive regions from various inter-simple sequence repeats (ISSRs) are amplified from genomic DNA by multiplexed PCR with tailed ISSR primers (1st PCR). (**b**) The 1st PCR products are subsequently used as the templates for the 2nd PCR (tailed PCR). This step enables the addition of complementary sequences for the binding sites of Illumina sequencing flow cell and indices (barcodes) for each sample to the 1st PCR products, using common forward and indexed reverse primers. (**c**) After measuring the approximate concentration of each 2nd PCR product, they are pooled in equimolar concentrations as a single mixture library. The mixture is then purified, fragments with a size range of 300–800 bp are isolated, the final concentration is measured by quantitative PCR, and is then used for Illumina paired-end sequencing (reads 1 and 2) and index reading. Sequencing of the first 17 nucleotides (primer region) of read 1, and 3 nucleotides (anchor region) of read 2 are skipped using the ‘DarkCycle’ option of the sequencer (indicated as the gray region in the arrows). (**d**) The sequence of the resulting library consists of binding sites for the P5 flow cell oligonucleotides and read 1 sequencing primer, forward ISSR primer, DNA insert, reverse ISSR primer, binding sites for read 2 and index sequencing primers, and P7 flow cell oligonucleotides.

To construct highly reduced representation libraries, we applied two PCR steps: multiplexed PCR with tailed ISSR primers as the 1st PCR, and tailed PCR with common and indexed primers as the 2nd PCR (Fig. 1). We selected a set of ISSR primers representing all types of 12 possible bases of two- or three-base core SSR sequences, with two-base anchor sequences on the 3′ ends of the primers (see Methods). In addition, sequence tails were added that correspond to a portion (14 bases) of the Illumina adapter sequence and three bases of the anchor for the 2nd PCR primers to confer adapter-strand specificity according to the published design^18^. In total, the 1st PCR primers comprised 14 (5′ tail) + 3 (anchor for the 2nd PCR primers) + 12 (SSR) + 2 (anchor) = 31 bases. Based on the standard rule to design PCR primers, eight primers (forward and reverse primers have the same sequences except for their sequence tails) were selected as the most recommended set of MIG-seq primers (set-1) for the 1st PCR (Table 1). These primers amplified a considerable number of fragments from all DNA samples effectively in the present study. Although we used the 8 × 8 primers for the 1st PCR in the present study, we also designed an alternative set of MIG-seq primers (set-2) for the 1st PCR comprising 4 × 4 primers in case additional or different markers and more highly reduced representation libraries were needed (Supplementary Table 1).

Subsequently, the 1st PCR products were used as templates for the 2nd PCR (tailed PCR). Using common forward and indexed reverse primers, this step permits the addition of complementary sequences for the binding sites of Illumina sequencing flow cell and index (barcode) for each sample to the 1st PCR products. These PCR steps were conducted for each sample independently. We normally treated 96 (or ~192) samples with different indices for one MiSeq run using 96-well PCR plates. However, after measuring the approximate concentration of each 2nd PCR product, they were pooled in equimolar concentrations as a single mixture library. They were then purified, fragments in the size range of 300–800 bp were isolated, and their final concentrations were measured by quantitative PCR.

### Sequencing, filtering, SNP discovery and genotyping

The library was subsequently used for sequencing on an Illumina MiSeq Sequencer. Both ends of the fragments were read by paired-end sequencing (reads 1 and 2), and 50 million sequence reads were detected in one run (*ca*. 500 K reads per sample in the 96-plex protocol).

Raw reads from each indexed sample were grouped together using the index reads option of the sequencer. After trimming the primer region, excluding the low-quality and short reads with adapter sequences, only 80-nucleotide sequences from both ends of the fragments (reads 1 and 2) were used for SNP discovery. To identify and genotype SNP loci, we applied a software program, Stacks v. 1.15^19^. Briefly, the output data from each sample were grouped into putative loci (stacks), polymorphic sites were identified, and the putative loci were grouped together across samples. The genotypes at each putative locus in each individual were then determined based on several criteria (see Methods) and converted into a set of output files, depending on the following analysis.

### Mendelian inheritance of discovered variants (Analysis 1)

Variants from heterozygous loci are expected to segregate with a 1:1 ratio in haploid megagametophytes from a coniferous mother tree. To validate the reliability of the variants as genetic markers discovered by the MIG-seq method, we demonstrated Mendelian gametic segregation of the discovered variants in 16 megagametophyte tissues from a coniferous mother tree of *Picea abies* planted in the Kawatabi Field Science Center, Tohoku University (38°44′N, 140°45′E). After filtering the raw reads data, 13,336,172 reads were detected from 16 megagametophyte samples and a set of replicates from a leaf sample of the mother tree. The reads were grouped into 5,063 putative loci (stacks) across the samples. Most of the putative loci (4,010, 79%) were detected in fewer than 10 megagametophyte samples or in one of the replicate samples from the mother tree tissue. These putative loci were eliminated, and the remaining 1,053 putative loci were used for subsequent analysis. After eliminating 661 monomorphic loci (63%), the remaining reads were grouped into 392 putative SNP loci (stacks) across the samples. Then, 172 putative SNP loci (44%), of which the two replicates of the seed parent had concordant heterozygote SNPs, were selected and used for subsequent procedures.

The 1st PCR primers could occasionally anneal to adjacent positions on the right annealing sites, because of the redundancy of the ISSR regions. To eliminate pseudo-loci originating from this shifted priming, which were identified as different stacks by the software, homology searching was conducted to identify them. To simplify this step, SNP marker groups with >60% sequence homologies were simply eliminated from the analysis as possible pseudo-loci. We found 98 possible pseudo-loci probably including ‘true’ independent loci within the 172 putative SNP loci that showed >60% sequence homology, and the remaining 74 putative SNP loci (43%) were used for subsequent analysis.

A chi-square test was conducted for 47 out of the 74 loci for which haplotypes were detected in at least 10 samples and that did not have heterozygous SNPs in the haploid samples. However, only 43 loci were identified as unique loci after eliminating four putative loci that originated from one side of the paired-end fragments based on the linkage analysis and paired-end information in the raw data. Most of the putative loci conformed to the expected 1:1 segregation ratio, based on a chi-square test. Although significant deviations (0.05 > *P* > 0.01) were observed in four putative loci (4/43 = 9.3%), both putative alleles (variants) were detected in at least three megagametophyte samples (Supplementary Table 2).

Sequences of PCR products may include errors introduced by the PCR; therefore, we estimated the number of possible pseudo-variant loci originating from PCR errors. In the case of variant discovery at an individual level, it is possible to identify the errors using the genotype data from a pair of replicates of one sample. If variants were detected between the replicates in a locus, the locus should simply be eliminated from the subsequent analysis. In our replicates from one parent tree, we detected six variants within 83 putative loci (6/83 = 7.2%) as PCR errors.

Moreover, duplicated loci could be included in the putative loci and could cause over- or under-estimates of the results. We identified the possible duplicated loci within the putative loci by checking the genotype in haploid megagametophytes. Three types of duplicated loci were identified in 19 putative loci based on the genotyping information of heterozygote SNPs in the haploid samples: 1) Four putative loci were identified as duplicated loci of the type homo + homo with different alleles to each other (e.g. A/A and T/T). This type was characterized by appearing as a ‘heterozygote’ in all haplotype samples. 2) Eight putative loci were identified as the type homo + hetero (e.g. A/A and A/T). This type was characterized by showing 1:1 segregation of the ‘homozygote’ and ‘heterozygote’ in the haplotype samples. 3) Seven putative loci were identified as the type linkage (e.g. AA/TT, AT/AT and AT/TA). This type was characterized by showing a low frequency (1–3 samples) of the one type (‘heterozygote’ or ‘homozygote’) in the haplotype samples. No locus was observed as the type hetero + hetero (unlinked) (e.g. A/T and A/T) showing 1:2:1 segregations.

### Clone (individual) identification (Analysis 2)

**Figure 2:**
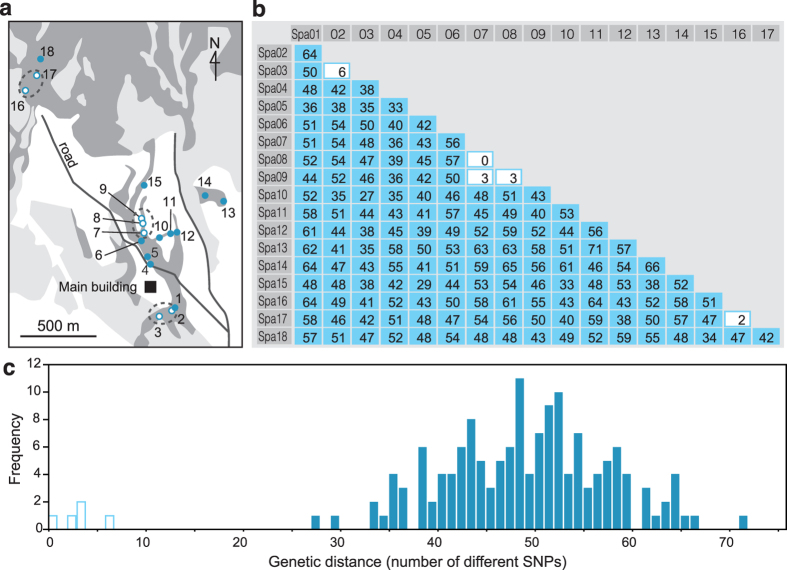
An example of clone identification by MIG-seq analysis. (**a**) Spatial distribution of 18 ramets (samples) and three clonal groups (genets, enclosed by dotted line) of *Sasa palmata* in the Kawatabi Field Science Center, Tohoku University (the main building is indicated on the map), inferred from 144 SNP markers discovered by MIG-seq analysis. Circles indicate the sampling site of each ramet in the distribution area (dark gray) of the species. White and light-gray areas indicate cultivated fields and conifer plantations, respectively. White and blue circles indicate ramets in an inferred clone and ramets from different clones, respectively. The map was generated by YM using Adobe Illustrator CC 2015 version 19.1.0 (Adobe Systems, San Francisco, CA, USA). (**b**) Matrix of the differences between pairwise numbers of SNPs in the 144 SNP markers among 18 samples of the species. White and blue columns indicate pairs of inferred clones with a small number of different SNPs (0–6) and ramets from different clones with large numbers of different SNPs (27–71), respectively. (**c**) Histogram showing the frequency distribution of the differences between the pairwise numbers of SNPs in the 144 SNP markers among 18 samples of the species. White and blue bars show the first peak represented by pairs of inferred clones, and the second peak is represented by pairs of ramets from different clones, respectively.

To validate the reproducibility of the genotyping data generated by MIG-seq, we conducted clone identification based on the genotypes of samples from both intra- and inter-clones of a clonal plant. We collected leaf samples from 18 ramets (culms) in a wild population of *Sasa palmata* from the Kawatabi Field Science Center, Tohoku University (Fig. 2a). Genotyping of the 18 samples was also conducted using seven microsatellite markers^20^,^21^ in advance.

We constructed a MIG-seq library for the samples, and sequenced them according to our standard protocol. After filtering the raw reads data, 10,472,792 reads were detected from the 18 samples in total. The reads were grouped into 3,665 putative loci (stacks) across the samples. In the case of population genetic samples (including Analysis 3), we simply eliminated the possible pseudo-variant (PCR error) loci having minor variants that were detected only in one sample, because random errors could not be shared among samples. We eliminated these artificial variants, which inevitably contained some possible ‘true’ variants, because, in general, low-frequency variants are less informative and cannot be treated as polymorphisms ( >1% frequency).

After eliminating less suitable loci, such as monomorphic loci, the genotypes of 144 SNP markers were used for clone identification. Using the GENODIVE^22^ program, genotypes from all samples were compared and the numbers of common and different SNP markers were calculated between all pairwise samples (Fig. 2b). Within the 18 samples, three multi-sample groups with nearly identical SNP genotypes were identified and the groups comprised two to three adjacent ramets (Fig. 2a). Although differences within the groups were detected for zero to six SNPs, the differences were clearly close to zero in the histogram of the frequency distribution of the differences between pairwise numbers of SNPs (Fig. 2c). The remaining 11 samples showed unique genotypes, with 27–71 different SNPs among them. These results agreed with the clone identification based on the microsatellite analysis (Supplementary Table 3).

### Population genetic analysis (Analysis 3)

**Figure 3:**
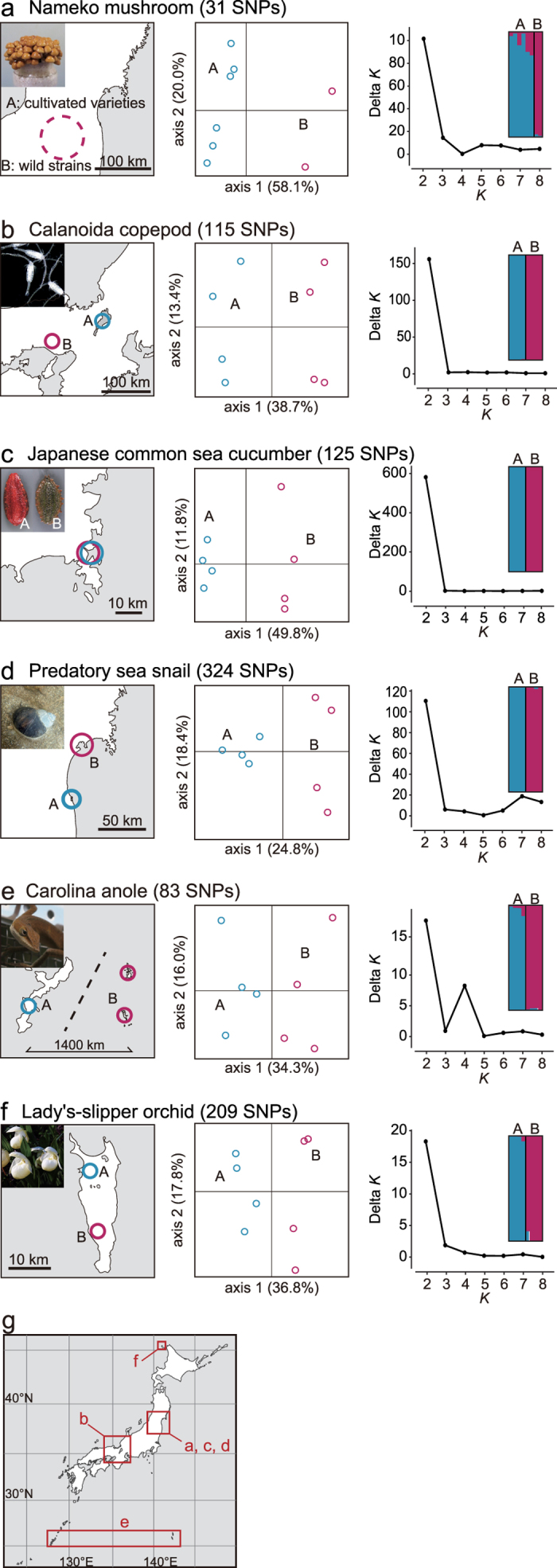
Examples of population genetic analysis based on MIG-seq for eight samples from two different populations (sources) each of six species: (**a**) nameko mushroom (*Pholiota microspora*); (**b**) Calanoida copepod (*Eodiaptomus japonicus*); (**c**) Japanese common sea cucumber (*Apostichopus japonicus*); (**d)** predatory sea snail (*Laguncula pulchella*); (**e**) Carolina anole (*Anolis carolinensis*); (**f**) lady’s-slipper orchid (*Cypripedium macranthos* var. *rebunense*). Sampling locations (left), plots of principal coordinate analysis (center), estimation of the optimum number of clusters (*K*, right), and proportion of the membership coefficient of eight samples in the STRUCTURE analysis (upper right) are shown. (**g**) Their sampling areas on a large-scale map are also shown. The map was based on the blank map available from Geospatial Information Authority of Japan and modified by YM using Adobe Illustrator CC 2015 version 19.1.0 (Adobe Systems). Photos by Manami Kanno (**a**,**c**), Wataru Makino (**b**), Jotaro Urabe (**d**), and Yoshihisa Suyama (**e**,**f**).

Using a standard population genetic analysis for a wide variety of species, we demonstrated the applicability of the method to a wide range of population genetic studies. We prepared eight samples from two different populations (sources) each of six species; 1) nameko mushroom (*Pholiota microspora*); 2) Calanoida copepod (*Eodiaptomus japonicus*); 3) Japanese common sea cucumber (*Apostichopus japonicus*); 4) predatory sea snail (*Laguncula pulchella*); 5) Carolina anole (*Anolis carolinensis*); and 6) Lady’s-slipper orchid (*Cypripedium macranthos* var. *rebunense*). We constructed MIG-seq libraries and sequenced them according to our standard protocol. After selecting suitable loci (see Table 2), 31–324 polymorphic putative loci were discovered in each species and used for subsequent standard population genetic analysis. Principal coordinate analysis and Bayesian clustering using STRUCTURE^23^ showed the expected genetic differentiation between the two populations in each species (Fig. 3).

## Discussion

MIG-seq is a PCR-based NGS method capable of constructing highly reduced representation libraries, discovering SNPs and genotyping them for multiplexed barcoded samples. MIG-seq offers an efficient approach, especially for medium-scale (~1,000 SNPs) marker-assisted genetic studies. Our recommended set of MIG-seq primers for the 1st PCR amplified thousands of regions effectively from various genomic DNA samples and permitted the discovery of tens to hundreds of SNPs in a wide variety of the tested species, although the numbers depend on the materials and conditions. The reliability of the genotyping was demonstrated by the expected Mendelian segregation in haploid megagametophytes and clone identification of a clonal plant. Furthermore, the utility of this method for standard population genetic studies was also demonstrated for six different species.

We considered five types of possible artifacts in the analysis: 1) Sequencing errors in the sequencing step. These errors were excluded through the data analysis step. Sequencing errors should appear only in one read as a random error and were eliminated through the SNP detection step in the program under the setting of minimum depth of coverage required to create a stack (m) = 20 and threshold for the minimum number of reads required to call heterozygous = 1/20 (if the ratio of the depth of two alleles is smaller than 1/20, the minor allele is rejected from the genotype). 2) PCR errors (pseudo-variants) were eliminated as minor variants that were detected only in one sample in the case of population genetic samples. In our replicates test (Analysis 1), six out of 84 SNPs (7%) might have originated from PCR errors. The causes of discovered variants between the replicates can be divided into two possibilities, sequencing and PCR errors. However, the sequencing errors were already eliminated through the SNP detection step as described above. By contrast, we found signs of PCR errors that could show a biased ratio of the read depth between each variant. Four out of the six variants showed a statistically different read depth from the 1:1 ratio based on a chi-square test (*P* < 0.05, data not shown). They could simply be eliminated from the analysis. 3) Pseudo-loci originating from shifted priming from an essentially identical locus might be a specific artifact in this method. To simplify the data analysis step, we eliminated all putative loci from SNP marker groups with >60% sequence homology as possible pseudo-loci; however, ‘true’ independent loci could be included in the eliminated markers. Further improvement of this step is required to avoid wastage. 4) Duplicated loci could be identified as a single locus in the detected markers and could cause over- or under-estimates of genetic differentiation. By comparing the genotype of a parent tree and its megagametophytes, we found three types of possible duplicated loci. These duplicated loci may not affect the essential results in studies to detect genetic differentiation among samples (or populations), but would directly affect the standard estimates for population genetics, such as heterozygosity. To avoid this risk, the duplicated loci should be eliminated after checking the Hardy-Weinberg equilibrium in a standard population, especially for population genetic studies that need exact estimates of genetic parameters. 5) Paired-end loci originating from both ends of one PCR fragment can be found as tightly linked markers using linkage (disequilibrium) analysis and paired-end information in the raw sequencing data. The paired loci may not affect the essential results in studies to detect genetic differentiation among samples, but they should be eliminated in the case of population genetic studies.

There is some room for improvement in our protocol. As the first example, because the numbers of amplified reads were quite different among the primers used for the 1st PCR (see Supplementary Figure 1), it might be possible to improve the amplification performance by modifying the primer set. Second, in the present intraspecific-level analysis, we did not apply presence or absence polymorphisms as dominant markers, but used SNPs in fragments amplified from the majority of the samples as co-dominant markers. It would be possible to apply the dominant markers, especially for interspecific analysis. However, it should be noted that presence or absence polymorphisms could be caused not only by actual polymorphisms based on sequence variations, but also by redundancy of the PCR amplification under low annealing temperatures.

In our standard protocol, we treated 96 samples for one sequencing run on an Illumina MiSeq Sequencer, using MiSeq Reagent Kit v3 (150 cycle, Illumina), which normally resulted in hundreds of thousands of reads, thousands of putative loci, and hundreds of SNPs per sample. It is possible to detect 300-nucleotide-long sequences using a 600-cycle kit (Illumina); however, we optimized our protocol for cost and time efficiency. According to our standard protocol, the total reagent cost to genotype hundreds of SNPs is approximately 15 US dollars per sample (mainly for the MiSeq kit and reagents for qPCR), excluding the initial cost for the 1st and 2nd PCR primers, and takes 3 days, comprising 1–1.5 day(s) for library construction, one night (17 h) for sequencing, and 1 day for data analysis. In contrast, a 600-cycle kit protocol costs approximately 25 US dollars per sample and takes 5–6 days, but detects more SNPs.

This method is unsuitable for high-density SNP genotyping because of the highly reduced representation. Although the cost and numbers of reads per sample are almost the same as existing methods (e.g. ~20 US dollars and ~1 M reads per sample for ddRADseq^11^), the number of SNPs is fewer in our method (e.g. ~1,000 *vs*. ~100,000 SNPs), which means low efficiency in terms of the cost per SNP and sequencing effort. Another considerable disadvantage of this method could be that the discovered SNPs are limited to ISSR regions. We may need to pay attention to the mutation rate and their neutrality, which might be affected by the adjacent SSRs when we use the markers for evolutionary and population genetic studies. However, MIG-seq provides a quick (3 days), simple (two PCR steps), and economical (15 US dollars per sample) approach for SNP genotyping that is applicable to a wide range of DNA qualities and quantities from a wide variety of species. We expect that MIG-seq will become the technique of choice for a diversity of marker-assisted genetic studies and will contribute to the conservation of biodiversity in the near future.

## Methods

### Template DNA

Total DNAs were extracted using suitable methods for each species from various amounts of material ranging from 6 μg (Calanoida copepod) to 50 mg of each sample (Supplementary Table 4). Approximately 2–100 ng of DNA was used for the 1st PCR as template DNA. Strict quantification and equalization of the sample DNA was not applied because heterogeneous amplification among the samples could be adjusted at the multiplexing step after the 2nd PCR.

### Primer selection

Several steps were conducted to select the MIG-seq primers as follows: 1) For the two-base anchor sequences, only AC, AG, CC, GG, TC and TG combinations were selected, because C or G is suitable for the 3′ ends of the primers and CG/GC is not suitable because of reverse complementarity. 2) For the three-base core motifs (sequences) that comprised three different bases (e.g. ACT), all combinations (4 × 3 × 2 = 24) were considered as candidates, but half of them (reverse complement sequences) were treated as candidates for an alternative set. 3) For the two-base core motifs comprising two different bases, only AC/CA and their reverse complements TG/GT, as an alternative set, were considered as candidates, because AG/GA (polypurine repeat), TC/CT (polypyrimidine repeat), GC/CG (GC rich) and AT/TA (GC poor) are unsuitable for general primers. 4) In the same way, only AAC/CCA and their reverse complements TTG/GGT were considered as candidates for the three-base core motifs comprising two different bases. 5) After combining the selected anchors with selected core motifs (e.g. 5′-(ACT)_4_TG-3′), primers that had i) only repeated sequence (e.g. 5′-(AC)_6_AC-3′), ii) only one base difference from a simple sequence repeat (e.g. 5′-(AC)_6_AG-3′), iii) GC rich on three bases of the 3′ end (e.g. 5′-(AC)_6_CC-3′), iv) less than three different bases (comprising only two different bases) (e.g. 5′-(CA)_6_CC-3′), and v) a reverse-complement sequence on four bases of its 3′ end (e.g. 5′-(ACC)_4_GG-3′) were rejected as unsuitable. 6) After combining with the tail sequences (e.g. 5′-CGCTCTTCCGATCTCTG(ACT)_4_TG-3′), primers that had more than three bases of reverse complement of the 3′ end sequence in its own or reverse primer were rejected as unsuitable. Then, 7) after comparisons with ‘surviving’ candidates in each alternative set, suitable primers for multiplexed PCR that did not have a reverse-complement conflict between three bases of the 3′ end and another primer sequence were selected. There were several choices to avoid the conflict; therefore, it was possible to select other primers instead of our choices. As a result, 8 × 8 and 4 × 4 primers were selected for the MIG-seq primer set-1 (Table 1) and set-2 (Supplementary Table 1) as an alternative choice, respectively.

### Library construction

The 1st PCR step was performed to amplify ISSR regions from genomic DNA with MIG-seq primer set-1 (Table 1). Alternatively, MIG-seq primer set-2 was used to create a different library from the same sample set (Supplementary Table 1). The volume of the PCR reaction mixture was 7 μl, containing 1 μl of template DNA, 0.2 μM of each 1st PCR primers, 3.5 μl of 2 × Multiplex PCR Buffer (Multiplex PCR Assay Kit Ver.2, Takara Bio, Kusatsu, Japan), and 0.035 μl of Multiplex PCR Enzyme Mix (Multiplex PCR Assay Kit Ver.2, Takara Bio). PCR was performed under the following conditions: initial activation at 94 °C for 1 min; 25 cycles for normal-concentration DNA (> 10 ng/μl) or 27 cycles for low-concentration DNA samples (< 5 ng/μl) from Calanoida copepod (see Supplementary Table 4) of denaturation at 94 °C for 30 s, annealing at 48 °C for 1 min and extension at 72 °C for 1 min; followed by a final incubation at 72 °C for 10 min, using a GeneAmp PCR System 9700 (Thermo Fisher Scientific). The PCR products were visualized using a Microchip Electrophoresis System (MultiNA; Shimadzu, Kyoto, Japan) with the DNA-2500 Reagent Kit (Shimadzu). Note that the 1st PCR could amplify a variety of ISSR regions, including some mismatched priming sites, depending on the conditions, because we decided to apply a relatively low annealing temperature (48 °C in our recommended system) for the 1st PCR after checking several different temperatures (data not shown). This annealing temperature could be effective to amplify more regions.

The 2nd PCR was performed to add the complementary sequences for the oligonucleotides that coat the Illumina sequencing flow cell, annealing sites of DNA sequencing primers, and indices to the 1st PCR products. The sequences of the common forward and indexed reverse primers were: 5′-AATGATACGGCGACCACCGAGATCTACACTCTTTCCCTACACGACGCTCTTCCGATCTCTG-3′ and 5′-CAAGCAGAAGACGGCATACGAGATxxxxxxGTGACTGGAGTTCAGACGTGTGCTCTTCCGATCTGAC-3′, where “xxxxxx” denotes the six-base index (Supplementary Table 5). This PCR step was conducted independently to add individual indices to each sample using the common forward and indexed reverse primers. The six-base index was designed using the Barcode Generator by Luca Comai and Tyson Howell (http://comailab.genomecenter.ucdavis.edu/index.php/Barcode_generator). The 1st PCR product from each sample was diluted 50 times with deionized water and used as the template of the 2nd PCR. The 2nd PCR was performed in a 15-μl reaction mixture containing 3 μl of diluted 1st PCR product, 3 μl of 5 × PrimeSTAR GXL Buffer (Takara Bio), 200 μM of each dNTP, 0.375 U of PrimeSTAR GXL DNA Polymerase (Takara Bio), and 0.2 μM of common forward primer and individual reverse primer. The PCR conditions were as follows: 12 cycles of denaturation at 98 °C for 10 s, annealing at 54 °C for 15 s, and extension at 68 °C for 30 s. The concentrations of each 2nd PCR product (libraries) were measured using a Microchip Electrophoresis System (MultiNA, Shimadzu) with a DNA-2500 Reagent Kit (Shimadzu). The libraries from each sample, each with a different index, were then pooled in equimolar concentrations. To reduce the salt concentration, the mixed libraries were purified and the buffer was replaced with elution buffer using a QIAquick PCR Purification Kit (Qiagen, Venlo, Netherlands). Fragments in the size range of 300–800 bp in the purified library were isolated using Pippin Prep DNA size selection system (Sage Science, Beverly, MA, USA). The final concentration was measured using a SYBR green quantitative PCR assay (Library Quantification Kit; Clontech Laboratories, Mountain View, CA, USA) with primers specific to the Illumina system.

### Sequencing

Libraries were denatured using fresh NaOH (0.2 N) and mixed with 10% of Illumina-generated PhiX control libraries, according to Illumina’s protocol. Approximately 10 pM of the libraries were used for sequencing on an Illumina MiSeq Sequencer (Illumina), using a MiSeq Reagent Kit v3 (150 cycle, Illumina). Note that Illumina cluster generation algorithms are optimized to a balanced representation of A, C, G, and T nucleotides, and the sequence diversity of the first part of the sequencing is particularly critical for recognition of the cluster position in a flow cell. The first 17 bases of both ends of MIG-seq library (anchor and ISSR primer region) have biased nucleotides in the libraries; therefore, sequence reading of these nucleotides should be skipped to gain high-quality data. We skipped the sequencing of the first 17 bases of read 1 and three bases of read 2 (anchor region) using the ‘DarkCycle’ option of MiSeq system. We left the remaining 15 nucleotides in the reverse read (read 2), because the ISSR primer region can be used to gather information on which ISSR primers were used (see Supplementary Figure 1) and does not cause problems in read 2 because it has no phasing step. The procedure for setting the ‘DarkCycle’ is shown in Supplementary Figure 2. Moreover, to replace the default index list with the original indices, a new ‘SamplePrepkit’ file was created and the file name was added to the list of ‘Compatible Sample Prep Kits’ in the file of ‘Assembly.txt’ in ‘Applications’ folder in the Illumina Experiment Manager in MiSeq system (Supplementary Figure 3). Both ends of the fragments and index sequences were read by paired-end sequencing (reads 1 and 2) and index sequencing; 80, 94 (except ‘DarkCycle’), and six bases of sequences were determined as read 1, read 2, and the index read, respectively. In total, 180 bases were sequenced using a 150-cycle kit.

### Data pretreatment and quality control

Raw reads from each indexed sample were grouped together using the index reads option of the sequencer. The first 14 bases (12 bases of SSR region and two bases of anchor sequences in the 1st primers) of read 2 sequences were trimmed using the program ‘fastx_trimmer’ in the FASTX-Toolkit (http://hannonlab.cshl.edu/fastx_toolkit/). Sequences of read 1 and trimmed read 2 were then quality-filtered by the ‘quality_filter’ option of FASTX-Toolkit, using the settings of q = 30 and p = 40. To remove the reads derived from extremely short library entries, the sequence primer region in the sequences of read 2 (GTCAGATCGGAAGAGCACACGTCTGAACTCCAGTCAC) and that of the read 1 sequence primer (CAGAGATCGGAAGAGCGTCGTGTAGGGAAAGA) were searched in read 1 and the trimmed read 2 sequences, respectively, and the reads that had these sequences were removed using TagDust^24^. Data from reads 1 and 2 of each sample were stored in the same folder and treated as independent reads.

Note that because the difference between the forward and reverse primers is only their tail sequence, one particular fragment (locus) in the genome could be amplified in both the forward-reverse and reverse-forward direction and could be read as both reads 1 and 2. Moreover, because the size range of the library is 300–800 bp, the two 80-nucleotide reads (reads 1 and 2) cannot overlap within each fragment and could be treated as independent reads. If reads 1 and 2 were connected and treated as single reads using paired-end information, forward-reverse and reverse-forward amplicons would be identified as different stacks, which would cause difficulties in the analysis step. Contrastingly, because the paired loci from reads 1 and 2 could be easily found by linkage analysis, even at the final step of the analysis, we decided to treat the two reads as independent reads. In addition, theoretically half of the PCR products could be amplified by forward-forward or reverse-reverse primers, which cannot be used for Illumina sequencing (the forward-reverse end is needed for the subsequent Bridge PCR step before DNA sequencing reactions). Therefore, the final output data to the next step include 80-nucleotide sequences from both ends of 300–800-bp forward-reverse amplicons of various ISSR regions.

### SNP detection

The quality-filtered reads were then used as input data for SNP detection with Stacks v. 1.15^19^. First, using the ‘ustacks’ option, a set of identical reads was bundled together in a ‘stack’, and several of these stacks were merged to form putative loci with the settings: maximum distance between stacks (M) = 2, enable the deleveraging algorithm (d), and the removal algorithm (r). The minimum depth of coverage required to create a stack (m) was set as 20. Second, a catalog was created for all possible loci and alleles with the ‘cstacks’ option. The parameter ‘number of allowed mismatches between samples (n)’ was set as four. All stacks created by ‘ustacks’ were then matched against the catalog produced by ‘cstacks’, using the ‘sstacks’ option.

### Selection of SNP markers

Two parameters, minimum percentage of samples in a population (r) and minimum number of populations in a locus (p), and the ‘write_single_snp’ option in the program option ‘populations’ in the Stacks program should be selected, depending on the sample set. We chose the parameters as r = 0.5 and p = 1 or 2 corresponding to the number of analyzed populations in each analysis. The results of Analysis 2 using different r (0.5 or 0.75) and m (see above, 5 or 20) parameters produced essentially the same results (Supplementary Figure 4). Additionally, the ‘write_single_snp’ option was also used to select only the first SNP of each locus. If a locus could not be detected in a sample, the genotype in the sample was treated as missing data.

In our segregation ratio check data (Analysis 1), a set of replicates from a seed parent and 16 megagametophytes were treated as independent populations to easily detect SNP loci that are common to the parent and megagametophytes. The parameter of the option ‘populations’ was set as p = 2. The putative loci of which the two replicates of the seed parent had concordant heterozygote SNPs were used for subsequent procedures. Then, homology searching was conducted to eliminate possible pseudo-loci originating from the shifted priming of primers using CLC Genomics Workbench (CLC bio, Aarhus, Denmark). We simply eliminated all putative loci in the SNP marker groups with >60% sequence homology that were concordant with less than 16 bases shifts ((80–16–16)/80 = 0.6)). The remaining putative loci that were detected from at least 10 haploid samples were used for subsequent analysis. The SNP calling model in Stacks is a diploid model; therefore, haploid megagametophytes must show homozygous SNPs. The putative loci that had heterozygote SNPs in the haploid samples were eliminated for segregation analysis and used to categorize duplicated loci. A chi-square test was conducted for the remaining putative loci to test the segregation ratio of the SNPs. Linkage analysis was conducted to find the loci originating from both ends of one amplicon or that were tightly linked loci, using Haploview^25^.

For our clone identification data (Analysis 2), the parameter of the option ‘populations’ was set as p = 1. To eliminate pseudo-variant loci derived from PCR errors, the putative loci of which the minor allele was detected only in one sample were removed. We also eliminated SNP marker groups with >60% sequence homology as possible pseudo-loci groups. To simplify the analysis, we did not check the linkage disequilibrium to find paired-end markers among the SNP markers for clone identification. Using GENODIVE^22^, the number of common and different SNPs was calculated for all pairwise combinations of samples.

For our population genetic data (Analysis 3), the parameter of the option ‘populations’ was set as p = 1. The aim of this analysis was to determine whether the two populations were distinguished into two groups; therefore, the initial setting of the number of populations was p = 1. The pseudo-variant loci (PCR errors) and pseudo-loci (shifted priming) were removed using the same procedure as described above. However, we did not conduct linkage disequilibrium analysis because of the small sample sizes. Principal coordinate analysis was performed on a matrix of covariance values calculated from population allele frequencies using GENODIVE. In addition, STRUCTURE version 2.3.3^23^ was used to infer the number of genetic populations or clusters (*K*). Values of *K* = 1–9 were tested by running 20 simulations for each *K*, with 50,000 Markov chain Monte Carlo iterations following a burn-in period of 50,000, using the model with admixture and correlated allele frequencies. The most likely number of *K* was determined using the software STRUCTURE HARVESTER^26^. The replicate structure runs were combined by CLUMPP^27^, and the results were visualized using DISTRUCT^28^.

## Additional Information

**Accession codes**: Raw MIG-seq data are deposited at the DDBJ Sequence Read Archive (DRA) with accession numbers DRA003553 (Submission), PRJDB3910 (BioProject), SAMD00030058–SAMD00030141 (BioSample), DRX031564–DRX031647 (Experiment) and DRR034931–DRR035014 (Run).

**How to cite this article**: Suyama, Y. and Matsuki, Y. MIG-seq: an effective PCR-based method for genome-wide single-nucleotide polymorphism genotyping using the next-generation sequencing platform. *Sci. Rep.* **5**, 16963; doi: 10.1038/srep16963 (2015).

## Supplementary Material

Supplementary Information

## Figures and Tables

**Table 1:** Sequences of MIG-seq primer set-1 for the 1st PCR.

Name	Sequences (5′–3′)
Forward primers: (Tail + **anchor: CTG**) + SSR + **anchor**	
(ACT)_4_TG-f	CGCTCTTCCGATCT**CTG**ACTACTACTACT**TG**
(CTA)_4_TG-f	CGCTCTTCCGATCT**CTG**CTACTACTACTA**TG**
(TTG)_4_AC-f	CGCTCTTCCGATCT**CTG**TTGTTGTTGTTG**AC**
(GTT)_4_CC-f	CGCTCTTCCGATCT**CTG**GTTGTTGTTGTT**CC**
(GTT)_4_TC-f	CGCTCTTCCGATCT**CTG**GTTGTTGTTGTT**TC**
(GTG)_4_AC-f	CGCTCTTCCGATCT**CTG**GTGGTGGTGGTG**AC**
(GT)_6_TC-f	CGCTCTTCCGATCT**CTG**GTGTGTGTGTGT**TC**
(TG)_6_AC-f	CGCTCTTCCGATCT**CTG**TGTGTGTGTGTG**AC**
Reverse primers: (Tail + **anchor: GAC**) + SSR + **anchor**	
(ACT)_4_TG-r	TGCTCTTCCGATCT**GAC**ACTACTACTACT**TG**
(CTA)_4_TG-r	TGCTCTTCCGATCT**GAC**CTACTACTACTA**TG**
(TTG)_4_AC-r	TGCTCTTCCGATCT**GAC**TTGTTGTTGTTG**AC**
(GTT)_4_CC-r	TGCTCTTCCGATCT**GAC**GTTGTTGTTGTT**CC**
(GTT)_4_TC-r	TGCTCTTCCGATCT**GAC**GTTGTTGTTGTT**TC**
(GTG)_4_AC-r	TGCTCTTCCGATCT**GAC**GTGGTGGTGGTG**AC**
(GT)_6_TC-r	TGCTCTTCCGATCT**GAC**GTGTGTGTGTGT**TC**
(TG)_6_AC-r	TGCTCTTCCGATCT**GAC**TGTGTGTGTGTG**AC**

Underlined and boldface nucleotides denote tail and anchor sequences, respectively. The difference between the forward and reverse primer sets lies only in their tail sequences. SSR; simple sequence repeat.

**Table 2:** Characteristics of the six species analyzed for population genetics by MIG-seq.

Species	Collection sites		Total no. of input reads for Stacks (min.–max.)	No. of putative loci (Stacks)	No. of SNPs
	A	B			
Nameko mushroom (*Pholiota microspora*)	Cultivated varieties from Miyagi Prefecture	Wild strains from Fukushima Prefecture	1,814,441 (138,636–301,781)	505	31
Calanoida copepod (*Eodiaptomus japonicus*)	Takashima, Shiga N35°15′, E136°4′	Ono, Hyogo N34°50′, E134°53′	3,195,755 (273,699–494,513)	3,212	115
Japanese common sea cucumber (*Apostichopus japonicus*)	Red types from Onagawa, Miyagi N38°25′, E141°29′	Green types from Onagawa, Miyagi N38°25′, E141°29′	9,892,527 (998,736–1,497,056)	3,409	125
Predatory sea snail (*Laguncula pulchella*)	Matsukawa-ura, Fukushima N37°46′, E140°58′	Matsushima, Miyagi N38°19′, E141°8′	7,887,899 (560,753–1,655,007)	4,843	324
Carolina anole (*Anolis carolinensis*)	Naha, Okinawa N26°13′, E127°41′	Ogasawara Islands N27°5′, E142°11′	6,773,549 (389,990–1,230,227)	2,354	83
Lady’s-slipper orchid (*Cypripedium macranthos* var. *rebunense*)	Northern Rebun Island N45°24′, E141°0′	Southern Rebun Island N45°19′, E141°0′	3,869,894 (349,500–650,200)	3,852	209
