# Supplementary material for: MIG-seq: an effective PCR-based method for genome-wide single-nucleotide polymorphism genotyping using the next-generation sequencing platform
Source: Sci Rep. 2015 Nov 23;5:16963. doi: 10.1038/srep16963 (PMC4655332; doi:10.1038/srep16963)
Supplement: Supplementary Information [file srep16963-s1.pdf]

Supplementary information

MIG-seq: an effective PCR-based method for genome-wide single-nucleotide polymorphism genotyping using the next-generation sequencing platform

Yoshihisa Suyama[1,*] and Yu Matsuki[1]

[1]Tohoku University, Kawatabi Field Science Center, Graduate School of Agricultural Science, 232-3 Yomogida, Naruko-onsen, Osaki, Miyagi 989-6711, Japan

*suyama@m.tohoku.ac.jp

Supplementary Table 1. Sequences of MIG-seq primer set-2 for the 1st PCR

Name	Sequences (5′–3′)
Forward primers:	(Tail + **anchor: CTG**) + SSR + **anchor**
$(CTG)_4$AC-f	CGCTCTTCCGATCT**CTG**CTGCTGCTGCTG**AC**
$(GCT)_4$TG-f	CGCTCTTCCGATCT**CTG**GCTGCTGCTGCT**TG**
$(GTA)_4$TG-f	CGCTCTTCCGATCT**CTG**GTAGTAGTAGTA**TG**
$(GTT)_4$AG-f	CGCTCTTCCGATCT**CTG**GTTGTTGTTGTT**AG**
Reverse primers:	(Tail + **anchor: GAC**) + SSR + **anchor**
$(CTG)_4$AC-r	TGCTCTTCCGATCT**GAC**CTGCTGCTGCTG**AC**
$(GCT)_4$TG-r	TGCTCTTCCGATCT**GAC**GCTGCTGCTGCT**TG**
$(GTA)_4$TG-r	TGCTCTTCCGATCT**GAC**GTAGTAGTAGTA**TG**
$(GTT)_4$AG-r	TGCTCTTCCGATCT**GAC**GTTGTTGTTGTT**AG**

Underlined and boldface nucleotides denote tail and anchor sequences, respectively. The differences between forward and reverse primer sets lie only in their tail sequences. SSR; simple sequence repeat

Supplementary Table 2. Segregation of allelic variants, their goodness of fit to the expected 1:1 ratio and linkage groups for 43 putative SNP loci, in megagametophyte samples from a mother tree of *Picea abies*

Locus	Genotype of the mother tree	Observed segregation in megagametophyte samples		P value (Chi-square test) * $P < 0.05$		Linkage groups with complete linkage
		Allele-1	Allele-2			
15	A / G	6	10	0.317		
40	A / G	11	5	0.134		A
136	G / T	10	4	0.109		
276	C / T	9	7	0.617		B
277	A / G	5	11	0.134		
343	C / T	9	7	0.617		
353	C / T	7	8	0.796		
416	C / T	7	8	0.796		
433	C / G	6	10	0.317		
437	A / G	6	10	0.317		
560	C / T	7	9	0.617		
666	A / T	7	9	0.617		
718	C / T	10	6	0.317		
756	C / T	12	4	0.046	*	
776	A / G	8	8	1.000		
923	A / C	6	9	0.439		
993	G / T	13	3	0.012	*	C
1005	A / G	9	6	0.439		
1048	A / G	5	11	0.134		D
1086	C / T	9	7	0.617		B
1118	A / C	9	7	0.617		
1311	A / G	8	8	1.000		
1368	A / G	5	10	0.197		A
1419	A / C	9	7	0.617		E
1428	C / T	5	11	0.134		D
1698	A / C	6	4	0.527		
1750	G / T	8	3	0.132		C
1768	A / G	12	4	0.046	*	
1771	C / T	8	8	1.000		
1853	C / T	7	9	0.617		
1856	G / T	4	6	0.527		
2025	C / T	7	9	0.617		
2054	A / G	9	7	0.617		E
2074	A / G	10	6	0.317		
2080	A / G	8	8	1.000		
2107	C / T	7	9	0.617		F
2113	C / T	7	9	0.617		F
2136	C / T	9	7	0.617		
2148	A / C	12	4	0.046	*	
2263	C / G	10	5	0.197		
2315	A / C	7	9	0.617		
2324	G / T	11	5	0.134		
2402	C / G	11	5	0.134		

Supplementary Table 3. Genotypes of seven microsatellite loci in 18 ramets (samples) of *Sasa palmata.* Three groups with identical genotypes (clones) are enclosed by a dotted line

	Locus													
Sample ID	*Sasa718*		*BWSS1*		*BWSS3*		*BWSS4*		*BWSS5*		*BWSS7*		*BWSS8*	
Spa01	161	161	109	109	140	140	93	97	75	75	97	97	109	109
Spa02	148	173	109	109	170	170	93	97	75	75	88	93	109	109
Spa03	148	173	109	109	170	170	93	97	75	75	88	93	109	109
Spa04	161	179	109	109	140	140	93	97	75	75	86	88	109	109
Spa05	150	165	105	105	140	170	101	103	75	75	88	88	109	109
Spa06	161	173	109	109	170	170	97	97	75	75	88	93	109	109
Spa07	171	173	109	109	187	187	97	103	75	75	88	97	109	109
Spa08	171	173	109	109	187	187	97	103	75	75	88	97	109	109
Spa09	171	173	109	109	187	187	97	103	75	75	88	97	109	109
Spa10	165	165	109	109	140	140	97	103	75	75	86	86	109	109
Spa11	161	163	109	109	140	170	95	101	75	75	88	88	109	109
Spa12	148	167	109	109	170	170	101	101	75	75	88	88	109	109
Spa13	161	163	109	111	140	183	97	103	75	75	88	95	109	109
Spa14	161	169	109	109	140	191	97	99	75	75	82	86	109	109
Spa15	161	165	109	109	140	140	95	95	75	75	88	88	109	109
Spa16	148	163	109	111	140	170	93	101	75	75	88	97	109	109
Spa17	148	163	109	111	140	170	93	101	75	75	88	97	109	109
Spa18	163	165	109	109	140	140	103	103	75	75	86	88	109	109

Supplementary Table 4. Description of DNA extraction for the eight species analyzed by MIG-seq

Species	Method (kit)	Amount of tissue used for DNA extraction (approx. mg, fresh weight)	Amount of DNA used as PCR template (ng)
Norway spruce (*Picea abies*)	DNeasy Plant Mini Kit (Qiagen)	0.01 (seed), 50 (leaf)	10–50
Dwarf bamboo (*Sasa palmata*)	CTAB method*	50	50–100
Nameko mushroom (*Pholiota microspora*)	Illustra Nucleon PhytoPure (GE Healthcare)	50	10–100
Calanoida copepod (*Eodiaptomus japonicus*)	GenElute Mammalian Genomic DNA Miniprep Kit (Sigma-Aldrich)	0.006 (dry)	2–3
Japanese common sea cucumber (*Apostichopus japonicus*)	QuickGene DNA tissue kit S (Kurabo)	5	10–20
Predatory sea snail (*Laguncula pulchella*)	DNeasy Blood & Tissue Kit (Qiagen)	0.6–2.1	10–20
Carolina anole (*Anolis carolinensis*)	Wizard Genomic DNA Purification Kit (Promega)	5	50–100
Lady's-slipper orchid (*Cypripedium macranthos* var. *rebunense*)	DNeasy Plant Mini Kit (Qiagen)	20–50	10–20

*Reference: Murray, M.G. & Thompson, W.F. Rapid isolation of high molecular weight plant DNA. *Nucleic Acids Res.* **8**, 4321–4325 (1980).

Supplementary Table 5. Sequences of six-base indices in the reverse primer for the 2nd PCR of MIG-seq

Index ID	Sequence	Index ID	Sequence	Index ID	Sequence	Index ID	Sequence
R001	AACATA	R097	AAAGTG	R193	AACATT	R289	AACGCC
R002	AACTAG	R098	AACCTG	R194	AACTAA	R290	AAGGAC
R003	AAGACT	R099	AATGTA	R195	AAGCCA	R291	AAGTGG
R004	AAGTTG	R100	ACACTG	R196	AATTCT	R292	ACAATT
R005	AATTGT	R101	ACCGAG	R197	ACCACG	R293	ACGAAT
R006	ACCCGA	R102	ACGACG	R198	ACCTGC	R294	ACTAGC
R007	ACCTCA	R103	ACGCTC	R199	ACGACA	R295	AGATCA
R008	ACGGCT	R104	ACGTGA	R200	AGACCT	R296	AGCCTG
R009	ACGTAT	R105	ACTATC	R201	AGCCGA	R297	AGGATG
R010	ACTACT	R106	ACTGAC	R202	AGGACT	R298	ATCATG
R011	ACTCGT	R107	ACTTAA	R203	AGGCGC	R299	ATGTTG
R012	ACTGCG	R108	AGATCT	R204	ATGGAG	R300	CAAGGA
R013	AGAACG	R109	AGATGC	R205	CAACGT	R301	CACCTT
R014	AGAAGT	R110	AGCAAT	R206	CACCGG	R302	CATTAG
R015	AGAGTT	R111	AGCGAC	R207	CATGTA	R303	CCATAG
R016	AGCCTT	R112	AGCTTG	R208	CCACTG	R304	CCGGAT
R017	AGCTCC	R113	AGTGAT	R209	CCGATC	R305	CCTCGA
R018	AGTAGG	R114	ATATAC	R210	CGACCA	R306	CGACTC
R019	ATACCG	R115	ATCACT	R211	CGCTGA	R307	CGAGCC
R020	ATATGG	R116	ATCCGG	R212	CGGTAG	R308	CGGACC
R021	ATCATC	R117	ATCGTG	R213	CGTTAA	R309	CGTGGT
R022	ATCCTA	R118	ATGCTT	R214	CTACTT	R310	CGTTGG
R023	ATGCAC	R119	ATGGTA	R215	CTCTTG	R311	CTGACG
R024	ATGGCC	R120	ATGTGC	R216	CTTATG	R312	CTTCAT
R025	ATGTAA	R121	ATTCTC	R217	GAACAC	R313	GACGAC
R026	ATTAGC	R122	ATTTCG	R218	GACCAT	R314	GAGTTC
R027	ATTGCT	R123	CAATTA	R219	GAGCGA	R315	GATCTC
R028	CAAGCG	R124	CACTCC	R220	GATTCC	R316	GCCTAG
R029	CACGCA	R125	CATAGG	R221	GCCGGA	R317	GCTCAA
R030	CATACA	R126	CATCTG	R222	GCTAGG	R318	GCTGTG
R031	CATCAT	R127	CATTGC	R223	GCTGAT	R319	GGACAT
R032	CCACAT	R128	CCAATG	R224	GGAGTT	R320	GGATAA
R033	CCCAAT	R129	CCACGA	R225	GGTATC	R321	GTAAGT
R034	CCGAGT	R130	CCGACA	R226	GTAGAG	R322	GTAGCA
R035	CCGTTG	R131	CCGCAC	R227	GTGTAC	R323	GTGTTA
R036	CCTGGC	R132	CCTACC	R228	GTTCCA	R324	TAACAA
R037	CGAGGT	R133	CGAAGG	R229	GTTGGA	R325	TACTCA
R038	CGATTT	R134	CGATAC	R230	TACGTG	R326	TAGCGG
R039	CGCCCT	R135	CGCAAC	R231	TAGCCT	R327	TAGTAT
R040	CGCTTC	R136	CGCGTT	R232	TCAACG	R328	TCATGC
R041	CGGATT	R137	CGGACG	R233	TCGGCT	R329	TCTACA
R042	CGTTCC	R138	CGTACT	R234	TCTTGT	R330	TGCCAA
R043	CTATCT	R139	CTACAA	R235	TGATTG	R331	TGCTGT
R044	CTCGAC	R140	CTATTC	R236	TGCGCA	R332	TGTAGA
R045	CTGATC	R141	CTCTCG	R237	TGGCTC	R333	TTACGC
R046	CTGGAT	R142	CTGCCA	R238	TTAACC	R334	TTAGCG
R047	CTGTGA	R143	CTGTAC	R239	TTATGA	R335	TTCAAT
R048	CTTGAA	R144	CTTACG	R240	TTGAAC	R336	TTGAGA
R049	GAACTC	R145	GAAATG	R241	AAGGCG	R337	AAGCAT
R050	GAAGAT	R146	GAATCG	R242	AATACG	R338	AATCGT
R051	GAAGCC	R147	GACTTG	R243	AATGGC	R339	AATGTT
R052	GAATGT	R148	GAGCCA	R244	ACATCG	R340	ACATTC
R053	GACACA	R149	GAGTGA	R245	ACCGCT	R341	ACCATA
R054	GACCGA	R150	GATCCT	R246	ACTAAG	R342	ACCTAT
R055	GAGATA	R151	GCAAGG	R247	ACTCTG	R343	ACGTGT
R056	GAGTAT	R152	GCCGGT	R248	AGCACA	R344	ACTGAA
R057	GATAAT	R153	GCGACT	R249	AGCGGC	R345	AGCATC
R058	GATTAA	R154	GCTCAT	R250	ATAGTG	R346	AGCTAC

R059	GCCATC	R155	GCTTAC	R251	ATGATA	R347	AGGCTT
R060	GCCTGC	R156	GGACCA	R252	ATTCCT	R348	ATGCCG
R061	GCTAGC	R157	GGATAG	R253	CAATCC	R349	ATTGCC
R062	GCTCTC	R158	GGCACT	R254	CACGAT	R350	CAATGG
R063	GCTTCT	R159	GGCTAT	R255	CATTGA	R351	CATGAC
R064	GGAGTG	R160	GGGTGC	R256	CCATGT	R352	CCAAGC
R065	GGATTC	R161	GGTGAC	R257	CCGTAA	R353	CCGAAG
R066	GGCCAC	R162	GTACGA	R258	CGAGAT	R354	CCTAAT
R067	GGCTGA	R163	GTATAA	R259	CGGCTA	R355	CGAATA
R068	GGTACA	R164	GTGAAG	R260	CGTATT	R356	CTAAGG
R069	GTAACC	R165	GTGCCT	R261	CTAACA	R357	CTAGCT
R070	GTAGCT	R166	GTTCTA	R262	CTCAAC	R358	CTCGAA
R071	GTATGC	R167	TAAACG	R263	CTGATT	R359	CTGCGT
R072	GTGATT	R168	TAACTG	R264	CTTCTA	R360	CTTGCA
R073	GTTAGT	R169	TACAAG	R265	GAAGGC	R361	GACTCT
R074	GTTGCA	R170	TACTTA	R266	GACGTA	R362	GATACT
R075	TAACGC	R171	TAGGTA	R267	GATAAC	R363	GATTAT
R076	TAATCC	R172	TATCGT	R268	GCACCG	R364	GCAAGA
R077	TACGAC	R173	TATCTC	R269	GCGGTA	R365	GCATTA
R078	TAGATC	R174	TATGCG	R270	GCTCGC	R366	GCGTGA
R079	TATACC	R175	TCCATT	R271	GCTTCA	R367	GCTCTT
R080	TATGGC	R176	TCCTAC	R272	GGCTCC	R368	GGAACT
R081	TCCCTA	R177	TCGCAT	R273	GTAATG	R369	GGACGA
R082	TCGAGG	R178	TCGGAG	R274	GTGCAA	R370	GGCTTA
R083	TCGCTG	R179	TCGTAA	R275	GTTACC	R371	GTACTA
R084	TCTATG	R180	TCGTGC	R276	GTTGCG	R372	GTGGCC
R085	TCTGGT	R181	TCTCGC	R277	TAATCT	R373	GTTCAC
R086	TCTTCG	R182	TCTGTA	R278	TACTGG	R374	TACCTC
R087	TGACAT	R183	TGAAGC	R279	TAGGTT	R375	TAGAGT
R088	TGAGTA	R184	TGAGGG	R280	TCCACC	R376	TCCGCG
R089	TGCACG	R185	TGATGT	R281	TCTCGG	R377	TCTGAG
R090	TGCTTT	R186	TGCGTG	R282	TGACGG	R378	TGAATC
R091	TGGTGA	R187	TGGACC	R283	TGCCGC	R379	TGATAT
R092	TTACAC	R188	TGTGCT	R284	TGCTTC	R380	TGCGAG
R093	TTCTCA	R189	TTAGCA	R285	TGTTCG	R381	TGGAGC
R094	TTGACT	R190	TTCCGC	R286	TTAGAC	R382	TGTTGC
R095	TTGCGT	R191	TTCGTA	R287	TTCGTT	R383	TTCTCG
R096	TTTACA	R192	TTGCTA	R288	TTGCTG	R384	TTGGCA

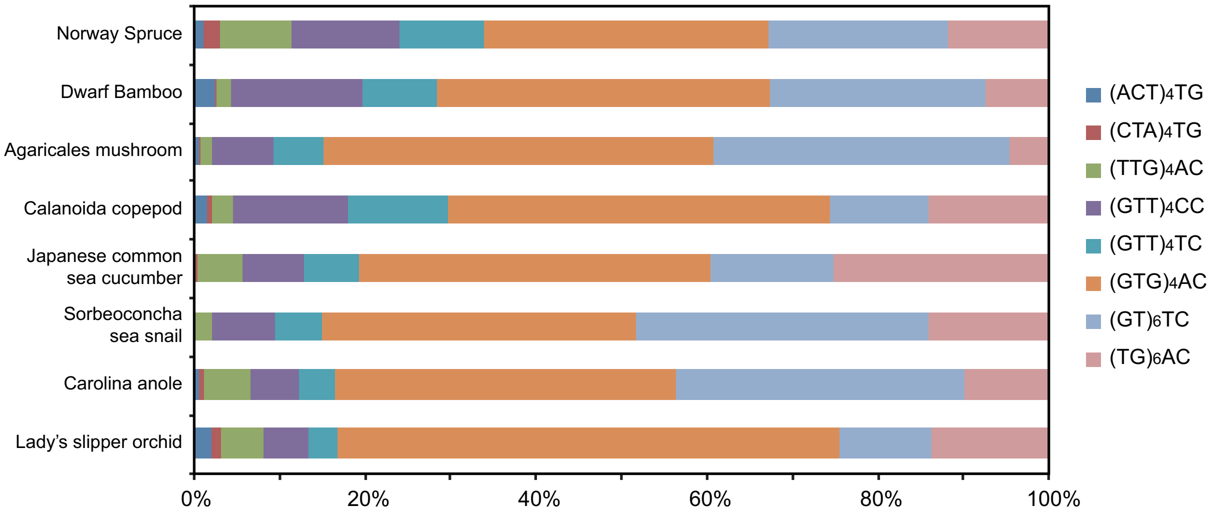

Supplementary Figure 1. Differences in the number of amplified reads among eight primers used for the 1st PCR

Copy and rename 'Amplicon' folder
in OSDisk(C)/Illumina/MiSeq Control Software/Recipe/V3/

OSDisk(C)/Illumina/MiSeq Control Software/Recipe/V3/

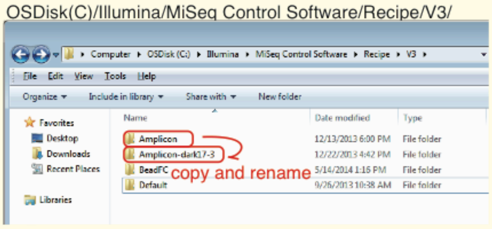

Modify the protocol of seqeunce reading program.

OSDisk(C)/Illumina/MiSeq Control Software/Recipe/V3/Amplicon-dark17-3/Protocol/**2Read1Index.xml**

Original "2Read1Index.xml" Modified "2Read1Index.xml" for DarkCycle

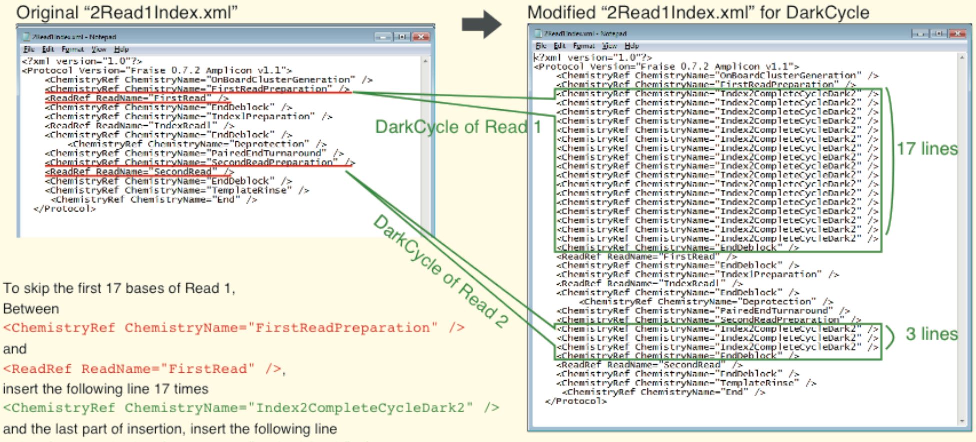

To skip the first 17 bases of Read 1,
Between

```
<ChemistryRef ChemistryName="FirstReadPreparation" />
```

and

```
<ReadRef ReadName="FirstRead" />,
```

insert the following line 17 times

```
<ChemistryRef ChemistryName="Index2CompleteCycleDark2" />
```

and the last part of insertion, insert the following line

```
<ChemistryRef ChemistryName="EndDeblock" />
```

To skip the first 3 bases of Read 2,
Between

```
<ChemistryRef ChemistryName="SecondReadPreparation" />
```

and

```
<ReadRef ReadName="SecondRead" />,
```

insert the following line 3 times

```
<ChemistryRef ChemistryName="Index2CompleteCycleDark2" />
```

and the last line of insertion, insert the following line

```
<ChemistryRef ChemistryName="EndDeblock" />
```

Creat a sample sheet as usual, then open it with text editor,
and change "Amplicon" to "Amplicon-dark17-3" on "Chemistry" line.

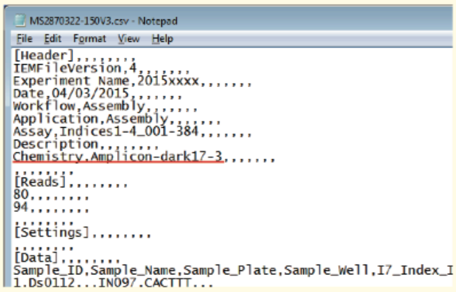

Supplementary Figure 2. Procedure for setting the DarkCycle in the MiSeq Control Software

Edit "**SamplePrepkits**" and "**Applications**" folders in OSDisk(C)/Program Files (x86)/Illumina/Illumina Experimental Manager/

1. In "SamplePrepkits" folder, copy an existing file ("TruSeq LT.txt" etc.).
   Change [Name] and [PlateExtension] column, and replace the index list of [P7].
   Save the file as the same name of [name] column.

Original "TreSeq LT.txt"

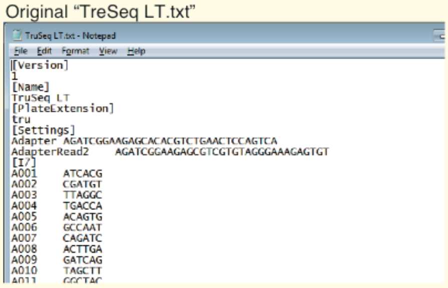

Modified file for original indices

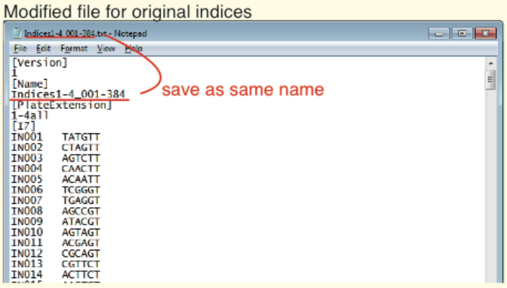

Caution: the sequence of listed index must be the reverse complement of index sequence of primer oligo.

Example: primer sequence

R001_AACATA:CAAGCAGAAGACGGCATACGAGATAACATAGTGACTGGAGTTCAGACGTGCGTGCTCTTCCGATCTGAC

Listed seqnece in index list TATGTT

2. Edit "Assembly.txt" in "Applications" folder.
   Add the modified sample prep kit to the list of [Compatible Sample Prep kits]

The added sample prep kit will be fould in Illumina Experimental Manager

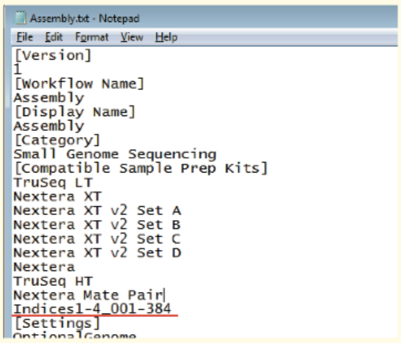

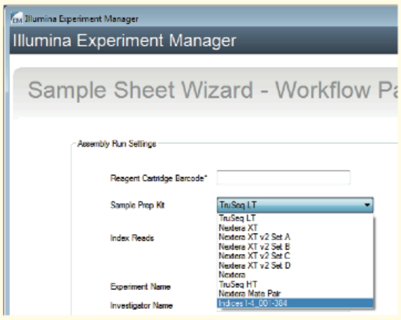

Supplementary Figure 3. Procedure for setting the original indices in the Illumina Experimental Manager in the program files of the MiSeq operation system

a

	Spa01	02	03	04	05	06	07	08	09	10	11	12	13	14	15	16	17
Spa02	137																
Spa03	110	5															
Spa04	101	106	97														
Spa05	85	85	72	63													
Spa06	94	101	96	85	71												
Spa07	113	118	96	88	95	110											
Spa08	120	116	92	92	95	111	8										
Spa09	79	89	73	65	76	81	6	2									
Spa10	114	107	98	80	76	79	111	123	93								
Spa11	130	117	97	97	101	112	124	122	92	117							
Spa12	122	95	67	87	78	111	102	110	78	107	129						
Spa13	121	95	86	85	77	81	113	113	81	86	104	103					
Spa14	123	107	83	116	90	105	139	135	95	146	118	112	108				
Spa15	95	90	73	78	64	87	101	103	77	85	109	98	96	110			
Spa16	126	108	99	91	95	90	94	100	82	108	120	116	94	124	90		
Spa17	109	92	80	74	83	68	80	86	71	92	101	89	73	96	78	6	
Spa18	140	118	87	109	88	105	95	105	79	111	109	119	105	127	100	102	83

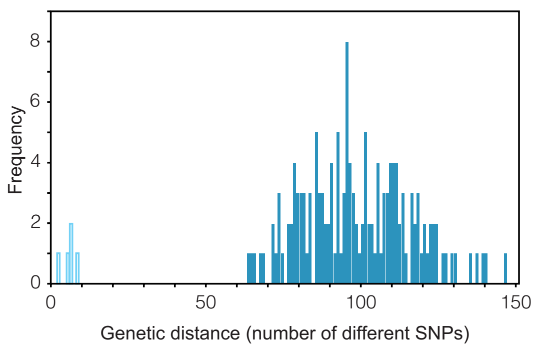

b

	Spa01	02	03	04	05	06	07	08	09	10	11	12	13	14	15	16	17
Spa02	78																
Spa03	70	3															
Spa04	70	68	65														
Spa05	68	67	62	54													
Spa06	68	77	71	67	59												
Spa07	76	76	75	73	72	81											
Spa08	81	80	78	77	75	85	2										
Spa09	69	66	63	59	65	71	3	2									
Spa10	68	80	78	64	63	75	83	87	82								
Spa11	69	72	68	74	75	80	82	87	76	75							
Spa12	81	67	58	68	63	81	74	78	65	70	90						
Spa13	80	67	65	61	56	60	77	80	63	63	75	79					
Spa14	73	73	63	80	68	79	85	93	83	98	74	84	81				
Spa15	66	55	54	62	58	62	72	80	68	64	72	69	62	75			
Spa16	89	75	74	76	72	66	78	83	77	86	86	81	74	96	63		
Spa17	85	71	66	62	64	60	67	72	65	75	77	70	62	86	54	6	
Spa18	84	64	60	82	78	80	67	76	71	84	79	77	62	80	61	65	59

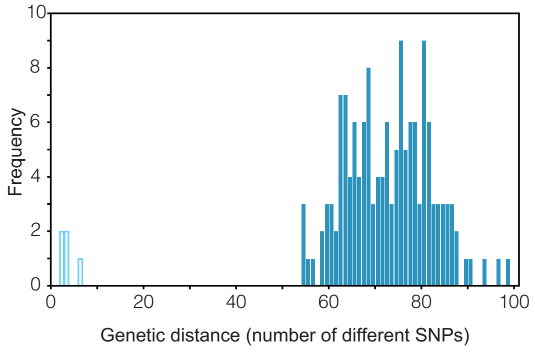

c

	Spa01	02	03	04	05	06	07	08	09	10	11	12	13	14	15	16	17
Spa02	40																
Spa03	37	3															
Spa04	30	33	33														
Spa05	32	35	32	30													
Spa06	47	44	40	28	39												
Spa07	31	38	37	30	37	45											
Spa08	35	41	39	36	40	50	0										
Spa09	30	39	37	29	37	43	1	1									
Spa10	31	25	21	24	38	37	28	32	27								
Spa11	34	30	27	28	28	48	34	39	34	37							
Spa12	34	27	27	32	36	39	35	42	38	28	35						
Spa13	44	29	27	39	36	43	42	44	43	32	44	41					
Spa14	36	33	30	38	33	40	35	38	34	35	33	32	30				
Spa15	33	33	31	31	25	33	39	43	36	24	28	37	26	35			
Spa16	48	38	35	34	35	40	36	41	35	35	43	32	38	35	36		
Spa17	48	36	35	35	38	40	36	40	33	33	42	31	38	37	37	2	
Spa18	38	29	29	32	39	40	28	30	27	27	34	37	24	34	22	27	27

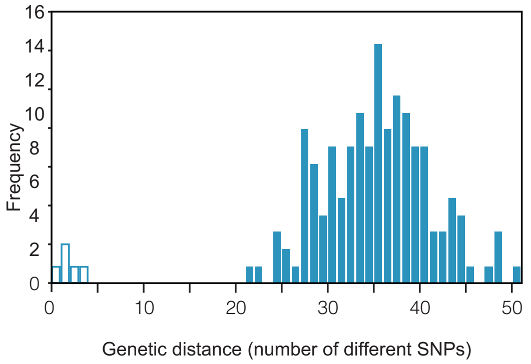

Supplementary Figure 4. Examples of clone identification by MIG-seq analysis based on different parameters (the minimum depth of coverage required to create a stack (m) = 5, 5 and 20; the minimum number of populations in a locus (r) = 0.5, 0.75 and 0.75 for **a**, **b** and **c**, respectively) used in the data analysis (see Figure 2 and Methods). Matrix of the differences between pairwise numbers of SNPs (left) and a histogram showing the frequency distribution of the differences between the pairwise numbers of SNPs (right) are presented.
